# Chiral nematic liquid crystal microlenses

**DOI:** 10.1038/s41598-017-01595-6

**Published:** 2017-05-09

**Authors:** Piotr Popov, Lawrence W. Honaker, Mona Mirheydari, Elizabeth K. Mann, Antal Jákli

**Affiliations:** 1 Physics Department, Kent State University, Kent, Ohio 44242 USA; 2 Liquid Crystal Institute, Kent State University, Kent, Ohio 44242 USA; 3 Physics and Materials Science Research Unit, University of Luxembourg, L-1511 Luxembourg, Grand Duchy of Luxembourg, Luxembourg

## Abstract

Nematic liquid crystals (NLCs) of achiral molecules and racemic mixtures of chiral ones form flat films and show uniform textures between circular polarizers when suspended in sub-millimeter size grids and immersed in water. On addition of chiral dopants to the liquid crystal, the films exhibit optical textures with concentric ring patterns and radial variation of the birefringence color. Both are related to a biconvex shape of the chiral liquid crystal film; the rings are due to interference. The curvature radii of the biconvex lens array are in the range of a few millimeters. This curvature leads to a radial variation of the optical axis along the plane of the film. Such a Pancharatnam-type phase lens dominates the imaging and explains the measured focal length of about one millimeter. To our knowledge, these are the first spontaneously formed Pancharatnam devices. The unwinding of the helical structure at the grid walls drives the lens shape. The relation between the lens curvature and material properties such as helical pitch, the twist elastic constant, and the interfacial tensions, is derived. This simple, novel method for spontaneously forming microlens arrays can also be used for various sensors.

## Introduction

Microlenses are a topic of considerable interest in applications ranging from biomimetic optical systems^1^ to security printing^2^ and solar concentrators^3^. The most commonly-used solid microlenses are made through delicate fabrication processes^4, 5^ and require mechanical adjustments to focus the image. In liquid lenses, the focal length is varied by electrowetting^6–10^. Liquid crystals (LC) can also be used to make lenses either by filling^11^ or imprinting LCs in curved substrates^12, 13^; by using a LC film with constant thickness, but spatially-varying refractive indices^14–16^; by creating focal-conic defects in smectics^17, 18^ and by using short pitch cholesteric liquid crystal polygonal textures^19^. The common feature of all these microlenses is the requirement for delicate fabrication processes. Thus, a simpler means of forming well-defined microlenses is highly desirable.

Carefully patterned LCs have been used to form Pancharatnam lenses. Unlike conventional phase or amplitude gratings, Pancharatnam phase^20^ devices operate by locally modifying the polarization state of light waves passing through them. Recently, the unique optical properties of Pancharatnam devices have been utilized in making high efficiency compact optical lenses by providing an appropriate profile across an aperture^21, 22^. In such lenses the optical axis is in the plane of the film with an azimuthal angle *β* that spatially varies along the radial direction.

Here, we demonstrate assembly-driven microlenses with size controlled by the grid in which chiral nematic liquid crystal films are suspended. Upon immersion in water, the suspended films spontaneously form converging spherical microlenses, which operate primarily as Pancharatnam lenses. We characterize the geometry and imaging properties of such lenses, and show that the lens shape is induced by molecular chirality and is driven by the liquid crystal-grid interaction.

## Materials and Methods

**Figure 1:**
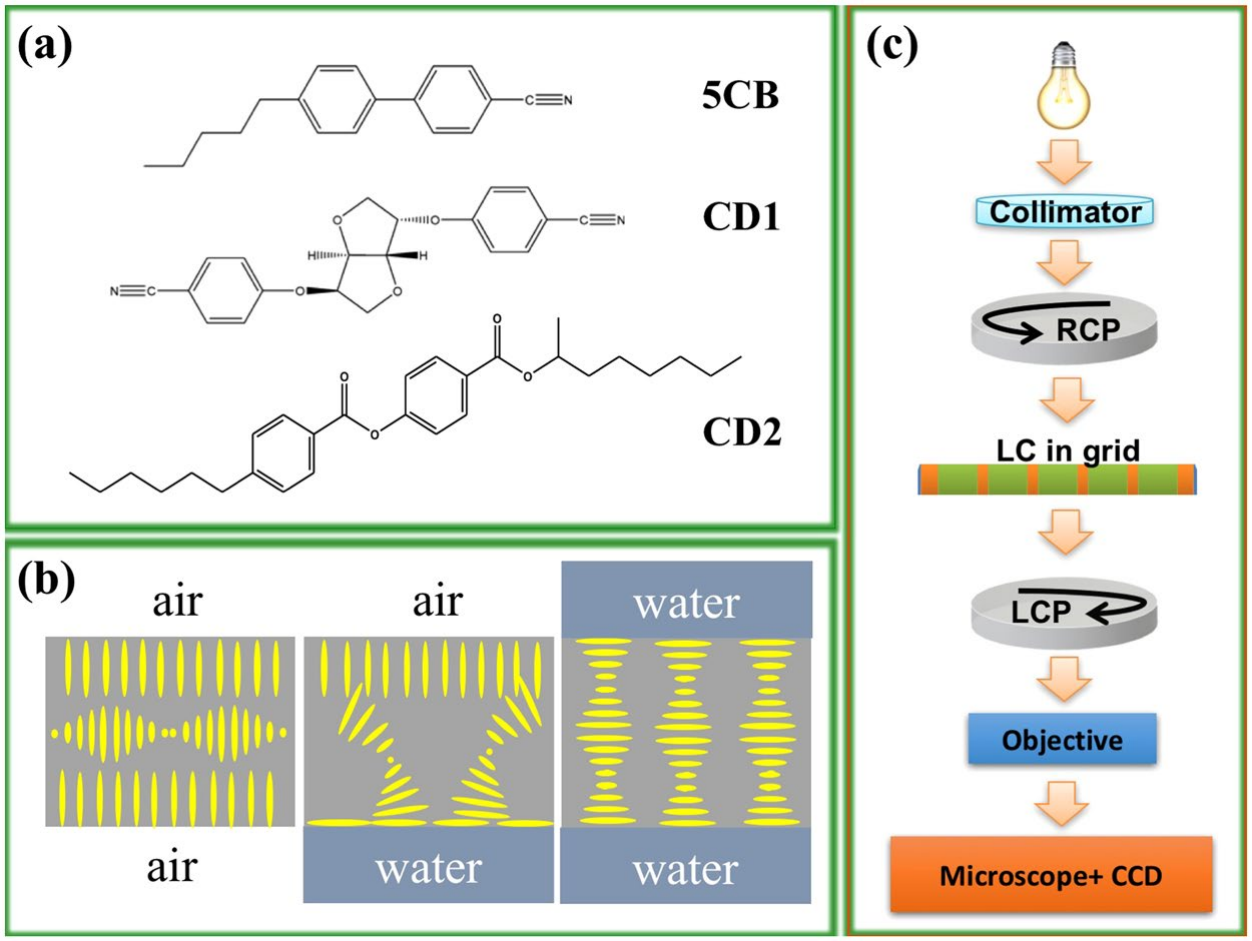
Studied materials and details of the experimental set-up. (**a**) Chemical structures of the host liquid crystal 5CB and chiral dopants CD1 and CD2, respectively; (**b**) Depictions of the configurations of the liquid crystal when bounded by air on both sides (homeotropic), water on one side and air on the other (hybrid), and water on both sides (planar); (**c**) Sketch of the experimental setup including the light beam, circular polarizers, a grid containing the LC film, and image acquisition using a CCD camera mounted on an inverted microscope.

The molecular structures of the studied materials and the experimental arrangements are shown in Fig. 1. Chiral nematic LC (N*) materials were obtained by adding chiral dopants CD1 or CD2 to the nematic LC (NLC) material 4-cyano-4′-pentylbiphenyl (5CB) obtained from Sigma-Aldrich and used without further purification. The nematic phase of 5CB is observed from 21.5 °C to 33.2 °C. Chiral dopant CD1 was obtained from Kent Displays, Inc., and (S)- and (R)-CD2 (code: ZLI 811 and ZLI 3786, respectively) were purchased from Merck.

To measure the helical twisting power (HTP) of the chiral dopants, a concentration gradient was created by putting the pure 5CB in contact with a c = 5 wt% mixture of *CD1* or *CD2* in *5CB*. Only integer numbers of the half pitch (p/2) can be accommodated within the cell thickness, d, so that bands with half-pitch jumps in twist form between the region with no dopant and the region with the full 5 wt%. Counting the number, k, of these bands yields the $HTP\equiv \frac{1}{p\cdot c}=\frac{k}{2d\cdot c}\,$. We found that *HTP* (*CD1*) ~ *6* *µm* ^−*1*^ and *HTP* (*CD2*) ~ *8* *µm* ^−*1*^. These mean that *5CB* + *5*% *CD1* has a pitch $p={(c\cdot HTP)}^{-1} \sim 3\,\mu m$, with uncertainties of about 20%.

The chiral mixtures were suspended in one half of a *d* = 20 µm thick 50/100 mesh nickel double grid from Ted Pella, Inc. Before each experiment, the grid and microscope dish were cleaned in methanol with an ultrasonic cleaner (Branson B200).

The LC-filled grid was manipulated by holding the unfilled half of the grid by tweezers and positioning it horizontally inside a dish that later was filled with water (Fig. 1c). The 35 mm glass-bottom microscope dish was obtained from Azzota. The water was purified by a PureLab Plus system (18.2 MΩ·cm). The presence of air or water sets the boundary conditions for the liquid crystal (Fig. 1b).

An Olympus CK40 inverted polarizing optical microscope (POM) was used to observe the N* films. A QICAM Fast1394 CCD camera was mounted on the microscope to capture images and videos. The sample was held either between circular polarizers with opposite handedness (see Fig. 1(c)) or between crossed linear polarizers. The intensity profiles of the N* films were analyzed by ImageJ™.

The interfacial tension between the nematic liquid crystal, with and without CD1, and water were measured with a homebuilt liquid droplet tensiometer: a droplet of the liquid crystal was extruded from a Hamilton syringe, fitted with a J-shaped needle, within a cuvette (borosilicate glass supplied by Wale Apparatus PA) filled with water. The droplet shape is determined by the balance of gravity and LC/water interfacial tension through the Laplace equation. The droplet shape was imaged by a CCD camera (PixeLink PL-B776F) with a light source (ThorLab) and diffuser (ThorLab). Before forming the droplet, the set-up was cleaned with methanol to remove any organic residues. The syringe and needle were each rinsed three times with chloroform. The cuvette was cleaned using KOH solvent (164 g MeOH, 24 g H_2_O and 25 g KOH), and rinsed with first deionized water and then ultra-pure water, a minimum of three times each. The drop shape was analyzed to determine the interfacial tension with Axisymmetric Drop Shape Analysis (ADSA) software provided by Applied Surface Thermodynamics Research Associates (ASTRA) from the Neumann group^23^. Interfacial tensions for oil/water interfaces agreed well with values found in the literature. For the liquid crystal droplet in water, the Bond (Eötvös) number was 0.3, within the range that this technique is valid; the uncertainty in the density difference between water and the liquid crystal was the main source of the measurement uncertainty. We found *γ* = (*9*.*4* ± *1*.*6*) *mN*/*m* for pure 5CB, consistent with literature values^24^, while for the 5CB/CD1 mixture *γ* = (*7*.*2* ± *1*.*4*) *mN*/*m*.

## Results

### LC films with different boundary conditions, chiral dopants, and grid size

**Figure 2:**
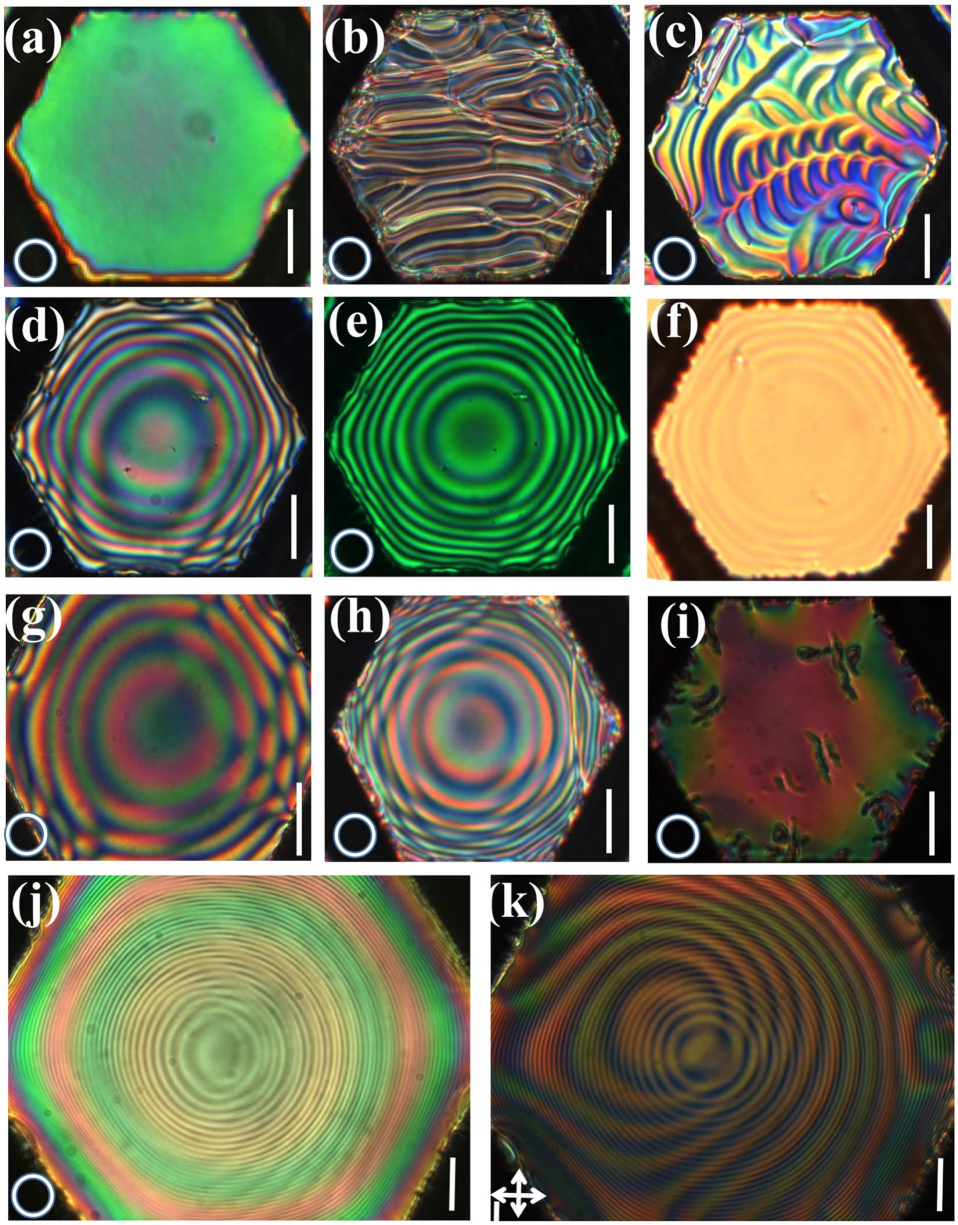
Polarizing optical microscopy pictures of LC films suspended in *d* = 20 *μm* thick hexagonal grids with various boundary conditions above and below the plane with 100 µm (**a**–**i**) and 215 µm (**j**,**k**) edge length. All pictures except for in (**f** and **k**) are taken between left-, and right-handed circular polarizers under white light illumination. (**a**) Pure 5CB between with water on both sides; (**b**) N* with 5% CD1 with air on both sides; (**c**) N* with 5% CD1 with air above and water below; (**d**–**f**) N* with 5% CD1 with water on both sides; (**e**) is the same as (**d**), but viewed with a green filter, and (**f**) is the same as (**d**) but without polarizers; (**g**) N* with 2.5% CD1 with water on both sides; (h) the same as (**d**) but with 3% CD2; (**i**) racemic mixture containing 1.5% (S)-CD2 and 1.5% (R)-CD2. (**j**) 5% CD1 in large grid between circular polarizers; (**k**) 5% CD1 between crossed linear polarizers. Bars correspond to 50 µm, while the circles in the lower-left hand corners indicates that the sample was between left and right handed circular polarizers while the crossed arrows in the same position indicates that the sample was between crossed polarizers.

Typical POM textures under white light illumination for various configurations of hexagonal grids with a = 0.1 mm sides are shown in Fig. 2. When the pure 5CB film is fully immersed in water and viewed between circular polarizers, one sees a nearly uniform color as shown in Fig. 2(a), demonstrating a uniform film thickness.

Figure 2 (b–f) shows textures of 5CB + 5 wt% CD1 (p ~ 3 µm). When the LC in N* phase is held in air, which provides homeotropic anchoring (molecules tend to align perpendicular to the film surfaces on both sides), a so-called “fingerprint texture”^25^ forms [see Fig. 2 (b)]. When the LC films in N* phase are put in contact with water from the bottom side (planar alignment) only, a hybrid alignment occurs resulting in a “ripple-like” fingerprint texture (see Fig. 2(c)). When the N* film is fully immersed in water (planar alignment on both sides), the fingerprint texture disappears and is replaced by colored circular patterns as seen in Fig. 2 (d). Note that the background birefringence color slowly changes between pale orange at the edge to green at the center. The Michel-Levy chart indicates a change of an optical path difference (Δ*n* · *d*) of about 300 nm. Alternating bright and dark rings stand out when a green filter is inserted after the light source [see Fig. 2(e)]. The rings are visible even without polarizers [Fig. 2(f)].

Figure 2(g–i) show similar ring patterns for two different CD1 concentrations and for another chiral dopant, CD2. The number of rings (i.e., the curvature) decreases with decreasing dopant concentration (i.e., with increasing helical pitch; Fig. 2(g)). The number of rings for 5CB with 3 wt% (S)-CD2 [Fig. 2(h)] with similar pitch is comparable to that of 5 wt% CD1, indicating that only the helical pitch, and not the specific chiral dopant, is important. Figure 2(i) shows the film when a nearly equal percentage of the (*S*) and (*R*) enantiomers of CD2 are added to 5CB, i.e., when the mixture is racemic. One sees no fringes, thus showing that an enantiomeric excess of chiral dopant is necessary for lensing.

Typical POM textures between circular and crossed linear polarizers for 5CB + 5 wt% CD1 liquid crystal suspended in hexagonal grids that are twice as large (edge length a = 0.22 mm) are shown in Fig. 2(j and k). In this case, the number of fringes is much larger (about 40) and the background birefringence varies over about three full bands (estimated change of optical path difference is about 1200 nm). In addition to the concentric stripes, off-centered Moiré-type stripes are also visible, especially when the sample is viewed between crossed linear polarizers.

### Lensing

**Figure 3:**
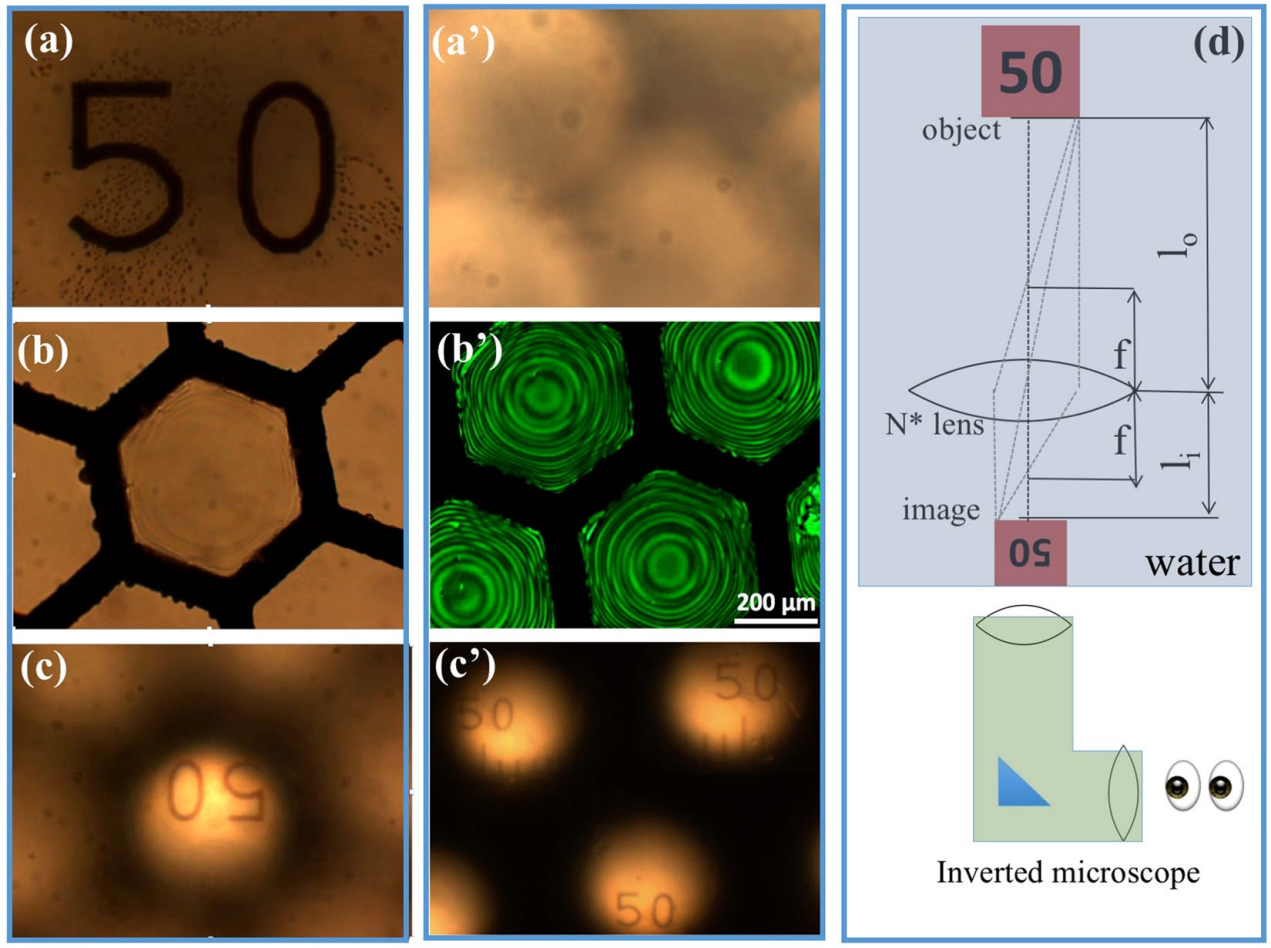
Demonstration of lensing using an N* microlens array. (**a**–**c**) micrographs seen when only the middle cell is filled with LC and the others are empty; (**a’**–**c’**) micrographs when every grid cells are filled with LC. (**a**) Direct image of a “50” marking on a microscope calibration slide, using light that travels through the empty cells. (**b**) Direct image of the N* microlens surrounded by empty cells. (**c**) Inverted image of the marking produced by N* microlens, using circular polarizer. (**a’**) “Image” seen at the equivalent position to (**a**), with all the grid cells filled with microlenses. (**b’**) Direct image of the array of microlenses as seen using a pair of circular polarizers and green filter. (**c’**) Array of inverted images of the marking produced by the N* microlens array, using circular polarizers. (**d**) Illustration of the geometric optics and the imaging setup for a single microlens, with the relevant parameters. Blue parts illustrate water medium, white part at the bottom illustrates the inverted microscope used to focus on the object, grid and image by changing the position of the objective. The different components are not to scale.

Our optical studies reveal that the suspended N* films immersed in water act as microlenses, i.e., can be used for image formation with visible light. This lensing effect is illustrated in Fig. 3.

The microscope calibration slide with a “50” marking placed between the light source and the N* microlens array (immersed in the water) was imaged by an inverted microscope. When only one of the grid cells was filled with LC (empty adjacent cells), the light could travel from the “50” marking to the microscope either through the lens or through the empty cells. Clear images of the marking were observed when light along either path was properly focused on the camera. The “50” marking of the slide could be seen with and without polarizers while its inverted image produced by the chiral nematic LC film could be seen only between polarizers (see also video in SI.) The objective lens must be in different positions to focus properly in these two cases. In one position, the direct marking was in focus [Fig. 3(a)]. Then, the N* LC lens itself was imaged by moving the objective by a distance *l*_*o*_ = *4*.*9* *mm* [Fig. 3(b)]. The inverted image of the “50” marking produced by the microlens was in focus after further moving the objective by *l*_*i*_ = *1*.*9* *mm* [Fig. 3(c)], with a 15% uncertainty. Assuming that *l*_*o*_ and *l*_*i*_ are the objective and image distances of the lens, the magnification of this lens would be *M* = *l*_*o*_/*l*_*i*_ = 1/2.6. This matches well with the apparent magnification of M_a_ = 1/2.7 obtained by comparing the sizes of “50” in Fig. 3(a) and (c). Small differences are expected since during focusing on the object, grid and image, the objective eyepiece length slightly varies, thus complicating the optics. Assuming that this complication is not significant, we estimate the focal length of the geometric lens with object and image immersed in water as ${f}_{G}=\frac{{l}_{o}\cdot {l}_{i}}{({l}_{o}+{l}_{i})}\approx 1.4\,mm$.

When the liquid crystal filled all the grids, no light path bypassed the microlenses and the marking could not be imaged directly [see Fig. 3 (a’)]. However, one inverted image appeared for each cell in the grid [see Fig. 3(c’)]. This is similar to image formation by the compound eyes of insects, except that the grids are flat in our case. The image of the suspended liquid crystal lenses are shown in Fig. 3(b’) using left and right circular polarizers and a green filter to clearly show the presence of the microlenses. The geometric optics are sketched in Fig. 3(d).

## Discussion

### Determining the lens shape

Since a water surface promotes degenerate planar alignment, the helical axis is expected to be perpendicular to the water/LC interface and the birefringence should be uniformly ${\rm{\Delta }}n={n}_{\parallel }-{n}_{\perp }$, where *n*_‖_ and *n*_⊥_ are the refractive indices along and perpendicular to the director. Such a film with uniform film thickness *D* would have uniform optical path difference $\xi ={\rm{\Delta }}n\cdot D$, and consequently uniform birefringence without the observed concentric rings. Thus, the rings must be due to varying film thickness, in contrast to the flat surfaces observed for the pure 5CB and the N* with homeotropic or hybrid alignments (Fig. 2(a–c)). The inverted image provided by the lens suggests that the effective lens shape is double convex and nearly spherical.

The different textural features might each have several origins. First consider the slow background variation in color: the observation of three distinct orders for the larger grids confirms that this is due to birefringence. The variation of the optical path difference $\xi ={\rm{\Delta }}n\cdot {\rm{\Delta }}D$ with Δn ~ 0.15 provides ${\rm{\Delta }}D \sim \frac{0.3\,\mu \,m}{0.15} \sim 2\,\mu m$ for the smaller grids with a = 0.1 *mm* and ${\rm{\Delta }}D \sim \frac{1.2\,\mu \,m}{0.15} \sim 7\,\mu m$ for *a* = *0*.*22* *mm*. The height *h* = Δ*D*/*2* and radius *a* of spherical caps can be related to the curvature radius *R* of the sphere as ${a}^{2}=h(2R-h)\approx 2Rh$ when *h* ≪ *a*, *i*.*e*., *R* = *a* ^2^/Δ*D*. This yields *R*~*5* *mm* for *a* = *0*.*1* *mm* and *R* ~ *6* *mm* for *a* = *0*.*22* *mm* size grids.

Next consider possible origins of the rings (particularly distinct as bright and dark rings under a green filter). They have some similarity to defect lines in Grandjean-Cano wedge cells^26^ marking jumps in number of half-pitches that can fit in the spatially-varying thickness. For this mechanism, the number of lines *m* is determined by the variation of the film thickness Δ*D* as ${\rm{\Delta }}D=m\cdot \frac{p}{2}$. For grids with a = 0.22 *mm*, we found *m* = *45* [see Fig. 2(j)], which would mean the lens would be *h* ~ *80* *µm* thicker in the middle than at the edges. Such an *h* is an order of magnitude larger than we estimated from the birefringent color and the constant volume requirement would result in negative thickness at the edge, i.e. they would not be stable. Additionally, due to the degenerate planar anchoring, the azimuthal angle of the director varies smoothly, which would be seen only between polarizers in contrast to rings that are visible even without polarizers (see Fig. 2(f)).

**Figure 4:**
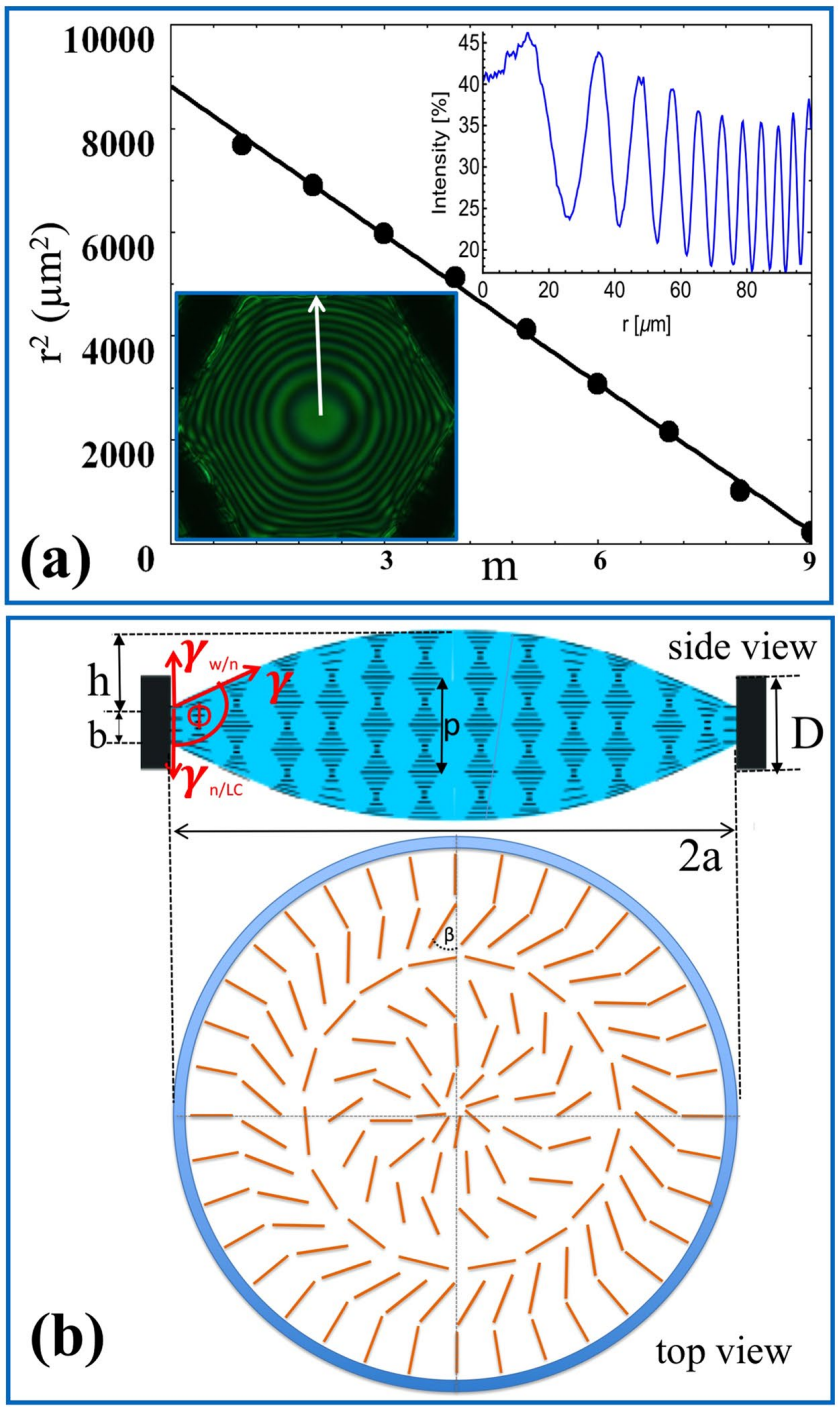
Analysis of the height profile of an N* LC microlens: (**a**) Plot of the square of the position of the minima with respect to their number, with m = 1 corresponding to the outermost ring. The linear dependence shows that the height profile is spherical. The lower inset shows the microlens viewed using a pair of circular polarizers and a green filter *λ* = 0.55 ± 0.02 *μm*. The length of the white arrow is 100 *μm*. The upper inset shows the intensity profile along the white arrow; (**b**) Cartoons of the directors within the cell: the upper images shows the cross sectional profile with relevant geometric and surface tension parameters, while the lower image shows the top view of the director configuration.

For these reasons, the rings are likely to be due to the interference between the liquid crystal film and water, as illustrated in Fig. 4(a). The position *r*_*m*_ of the *mth* dark Newton ring of a biconvex lens with volume *a* ^2^ *π*·*D*, height *h* and curvature radii *R* of both spherical caps can be approximated as

1 $${r}_{m}^{2}=R(D+h)-\frac{\lambda R}{2n}(m+1/2)$$

where λ = 0.55 µm is the wavelength of the light passing through the green filter and *n* is the refractive index of the lens (*n* = *n*_⊥_ ≈ 1.7). This shows that *r* ^*2*^ is proportional to m and the slope s of the line is $s=\frac{\lambda R}{2n}$. A plot of r^2^ vs. m is shown in Fig. 3(a). The fit to the line give s = 954 µm^2^, which provides R = 5 mm in agreement with the estimation from birefringence. This agreement confirms that the rings are due to interference between the water and the biconvex liquid crystal lens.

For symmetry reasons, and because of the density of LC is almost equal to that of the water, we can assume that the curvature radii of the spherical caps of the biconvex lens are basically equal at the top and bottom.

### Determining the lens focal length from the lens shape and director configuration

The lens shape deduced above implies a focal length given by (since the lens is much thinner than its radius) the Lensmaker equation^27^ $\frac{1}{{f}_{G}}\approx \frac{{n}_{l}-{n}_{m}}{{n}_{m}}\frac{2}{R}$. Here *f*_*G*_ is the focal length determined by the geometric optics; *n*_*l*_ is the average refractive index of the lens and *n*_*m*_ is the refractive index of the surrounding medium (water). With *n*_*m*_ = 1.33 and *n*_*l*_ ~ 1.7 (here, we assumed the extraordinary refractive index of 5CB + 5% CD is very close to that of the pure 5CB), we get ${f}_{G}=\frac{5\,mm\cdot 1.33}{2\cdot 0.37} \sim 9\,mm$. This is about five times larger than what we estimated from the imaging geometry. Additionally, microlens image formation could be detected in our experiments only between polarizers, indicating that the focal length is determined by the director configuration as well as the shape of the lens.

This situation is similar to imaging by smectic lenses. In that case, the focal length was dominated by the lateral variation of the refractive index^18^; in our case, the refractive index is basically uniform, equal to the extraordinary refractive index of the liquid crystal. On the other hand, due to the varying film thickness and the degenerate planar anchoring of water, the azimuthal angle of the director varies radially by *β*/2 = 2*π*Δ*D*/*p* from the edge to the center and by *β* over the diameter of the lens, Fig. 4(b).

A similar configuration was suggested for the corneal lenses of fireflies and June beetles^28,29^. These lenses are composed of lamellae, where different levels of laminae create spiraling ribbons in the thickness of the section. This spiraling structure is very similar to the centrosymmetric structure shown in Fig. 4(b), except at the center where spirals will avoid defects, whereas the concentric structure should lead to defects at the center. Based on the fact that no defects are seen in our experiments (see Fig. 2(d–k)), we conclude that the azimuthal rotation of the director is not completely centrosymmetric, but closer to the spiraling structure of the corneal lenses.The similarity between the structure of the corneal lenses of bug eyes and our cholesteric lenses suggest the self-assembly of the bug eyes is also driven by chirality.

Such a Pancharatnam-type phase lens has a focal length of *f*_*P*_ ≈ *πa* ^2^/(2*λβ*)^22^, where a is the radius of the lens, and *λ* is the wavelength of the light (in our case analyzed in, *λ* = *0*.*55* *µm*). Using the relations *β* = 4*π*Δ*D*/*p* and Δ*D* = *a* ^2^/*R* we can express the Pancharatnam focal length as

2 $${f}_{p}\approx \frac{p\cdot R}{8\lambda }$$

For *R* ~ *5* *mm* and *p* ~ *3* *µm* we get *f*_*p*_ ~ *3* *mm*.

The actual focal length is due to the combination of the geometric and Pancharatnam lenses; if we approximate the combined effect as due to two closely-spaced lenses, the focal length is the geometric mean, $f={f}_{G}\cdot {f}_{p}/({f}_{G}+{f}_{p}) \sim 2.2\,mm$. This value is slightly larger than what was estimated from the image and object distances. This could be due to the ~20% measurement uncertainty, and because Eq. (2) is strictly valid only for half-wave plates, which is not fulfilled in our case, thus modifying *f*_*P*_.

### Origin of lens shape

After understanding the optics of our N* LC lenses, what is left to explain is the reason for the formation of the lensing shape under water. The equilibrium shape of the film can be obtained by minimizing the free energy density with respect to the height *h* of the spherical cap.

The free energy *W* related to the shape change can be divided into three terms: *W* = *W*_*LC*_ + *W*_*i*_ + *W*_*w*_. *W*_*LC*_ is the distortion energy density taking account the director deformation (splay, twist and bend) related to the shape change; *W*_*i*_ is related to the energy increase of the interface; and *W*_*w*_ is the decreased wall energy due to a decrease of the film thickness in the immediate vicinity to the grid.

The energy due to the distortion of the liquid crystal director can be expanded as *W*_*LC*_ = *W*_*splay*_ + *W*_*twist*_ + *W*_*bend*_, where ${W}_{splay}=\frac{{K}_{11}}{2}{\int }^{}{(\overrightarrow{\nabla }\cdot \overrightarrow{n})}^{2}dV \sim \pi {K}_{11}{({\rm{\Delta }}\phi )}^{2}{D}_{avg}$, ${W}_{bend}=\frac{{K}_{33}}{2}{(\overrightarrow{n}\times \overrightarrow{\nabla }\times \overrightarrow{n})}^{2}6{a}^{2}{D}_{avg} \sim \frac{3{K}_{33}}{2}{(\frac{\Delta \phi }{{D}_{avg}})}^{2}{a}^{2}{D}_{avg}$, and ${W}_{twist}=\frac{{K}_{22}}{2}{(\frac{2\pi }{p}-\frac{2\pi }{{p}_{d}})}^{2}6{a}^{2}{D}_{avg}$. Here, ${D}_{avg}\cong 20\,\mu m$ is the average thickness of the convex N* film and ${\rm{\Delta }}\phi =\varphi -\pi /2$ is the change of director angle due to the splay and bend deformations associated with the lens shape with contact angle ϕ [see Fig. 4(a)]. Assuming the elastic constants of the doped 5CB are similar to those of the pure 5CB, i.e., *K*_11_ = 6.4 *pN* ^30^, *K*_22_ = 5.4 *pN* ^30^, and *K*_33_ = 13.8 *pN* ^30^, we get that *W*_*LC*_ ~ 10^−16^ *J*. In fact, the twist term is exactly zero since the pitch does not need to be deformed (p_d_ = p) due to the degenerate planar anchoring.

The twist term does contribute to *W*_*w*_ due to the uniform homeotropic anchoring at the wall that is incompatible with the helical structure far from the walls. The positive energy related to the unwinding of the helix near the wall is decreased by reducing the thickness of the LC-wall contact area by Δ*D* = h (taking into account the fixed volume of the lens; see Fig. 4(d)). Taking into account that the depth of the unwound region is proportional to the width of the LC-wall contact area *b* = *D-h*, ${W}_{w}=-\frac{{K}_{22}}{2}\cdot {(\frac{2\pi }{{p}_{0}})}^{2}\cdot 6a\cdot (D-h)\cdot h$. For *a* = *0*.*1* *mm* and *D* = *20* *µm* experiments indicate that *h*~*1* *µm*, which gives *W*_*w*_ ~ 10^−13^ *J*, i.e., three orders of magnitude larger than *W*_*LC*_.

The interfacial contribution to the energy has two terms. *W*_*i*1_ is due to the replacement of LC-wall interface by water-wall interface on an area of 6*a*·Δ*D* = 6ah. Denoting *γ*_*LC*/*N*_ − *γ*_*N*/*w*_ = Δ*γ*, where γ_LC/N_ and γ_N/w_ are LC-nickel and nickel-water interfacial tensions we get ${W}_{i1}=6{\rm{\Delta }}\gamma \cdot a\cdot h$. Utilizing that ${\rm{\Delta }}\gamma =-\,\gamma {\rm{c}}{\rm{o}}{\rm{s}}\varphi =\gamma {\rm{s}}{\rm{i}}{\rm{n}}{\rm{\Delta }}\phi$, where γ = γ_LC/w_ is the liquid crystal-water interfacial tension [see Fig. 4(b)] and taking into account that ${\rm{s}}{\rm{i}}{\rm{n}}{\rm{\Delta }}\phi =a/R=2h/a$, we get *W*_*i*1_ = 12*γh* ^2^. The second term of W_i_ is due to the increase of the LC-water interface area. Using spherical caps W_i2_ approximated as *W*_*i*2_ = 2*γπh* ^2^. Using the value for γ = γ_LC/w_ ~ 10 mN/m as discussed in the methods section, we find W_i_  ~ 10^−13^ *J*, which is similar to the magnitude of the wall energy.

Accordingly, *W*_*LC*_ can be neglected, and the height of the spherical caps can be determined by the condition $\partial ({W}_{w}+{W}_{i})/\partial h=0$. This yields

3 $$h\cong \frac{3{K}_{22}{(2\pi /h)}^{2}aD}{4\gamma (\pi +6)+6{K}_{22}{(2\pi /h)}^{2}a}\approx \frac{3{\pi }^{2}{K}_{22}aD}{\gamma {p}^{2}(\pi +6)}$$

Equation (3) shows that *h* (and therefore the curvature radius *R* = *a* ^*2*^/*2* *h*) depends on the twist elastic constant *K*_*22*_, the helical pitch *p*, the interfacial tension *γ*, and the size of the grid *a*.

Experimentally, the lateral size dependence is the easiest to check as other parameters can be kept constant. We found that h ~ 1 μ*m* for a = 0.1 *mm* and h ~ 3.5 *μm* for *a* = *0*.*22* *mm* grids, whereas Eq. (3) would predict an increase in *h* that is smaller by about 50%.

To check the prediction of Eq. (3) for the pitch (which should be inversely proportional to concentration) we can compare Fig. 2(d) and (g) that show m = 9 rings for 5% CD1 and m = 5 rings for 2.5% CD1. Assuming the refractive index *n* does not significantly change with the concentration, we find that *h*(*5*%)/*h*(*2*.*5*%) = *m*(*5*%)/*m*(*2*.*5*%)~*2*. On the other hand, from Eq. (3), assuming *K*_*22*_ and *γ* do not vary with the concentration of the chiral dopant, we would get *h*(*5*%)/*h*(*2*.*5*%) = *p* ^*2*^(*2*.*5*%)/*p* ^*2*^(*5*%) ~ *4*, which is about twice as large as the value obtained experimentally. Even if we take into account the 20% uncertainty of the determination of the concentration of the chiral dopants, this exceeds the experimental uncertainty.

The other parameters in equation 3 are more difficult to vary independently. To vary the anchoring (surface tension) on the grid, one either need to change the LC (which then will also change *n* and *γ*) or the grid material. We tested copper grids and found qualitatively similar behavior. However the copper grids were found to have very rough cell edges that introduced many defects. We also tested gold grids, which were difficult to hold with tweezers and would require a new type of sample holder. It would be interesting to coat grids with, for example, a hydrophobic layer; this technology is well-developed for gold, and is an interesting future direction. The remaining other parameter is the elastic constant, *K*_*22*_, which can in principle be varied with temperature. To do so requires a liquid crystal with a wider nematic temperature range and a new setup with precise temperature control. Of course, as the temperature and varies, so will *n* and *γ*, which should then be measured separately. All of these tests would therefore require a completely new set of measurements, which is beyond the scope of this paper.

However, we have demonstrated both that equation 3 is in agreement with the observed shape and that it is in qualitative agreement with the observed variations in shape with grid size and with pitch. However, *h* increases more than expected with grid size and less than expected with the concentration of chiral dopant. This discrepancy may be in part due to the fact that all other parameters (*K*_*22*_, *n* and *γ*) depend on the chiral dopant. However, we expect that the model needs to be refined based on a more in depth analysis.

In summary, we have demonstrated the spontaneous formation of converging spherical microlens arrays by immersing a chiral nematic liquid crystal suspended in sub-millimeter size grids into water providing an extremely simple method to make microlenses. We have also demonstrated the imaging capability of the microlenses and showed that the focal length is influenced by the combination of geometric optics and a spontaneously formed Pancharatnam optics. Finally, we have proposed a theoretical model that can explain the lensing effect and predict the dependence of the lens shape on material parameters (twist elastic constant and surface tension), and on sample geometry (thickness and lateral size of grids).

Although the model should be further tested experimentally and may need refining, the dependence of the shape on surface parameters means that presented lens array has a potential to detect the presence of nanoparticles or biologically relevant materials, similarly to other LC biosensors^31–33^.

## Electronic supplementary material

Video 1

Video 2

Supplementary Information
