# Supplementary material for: Chiral nematic liquid crystal microlenses
Source: Sci Rep. 2017 May 9;7:1603. doi: 10.1038/s41598-017-01595-6 (PMC5431631; doi:10.1038/s41598-017-01595-6)
Supplement: Supplementary file 3 — Supplementary Information [file 41598_2017_1595_MOESM3_ESM.pdf]

Supplementary Information

Chiral nematic liquid crystal microlenses

Piotr Popov[1], Lawrence W. Honaker[21], Mona Mirheydari[1], Elizabeth K. Mann[1], Antal Jákli[2*]

[1]Physics Department, Kent State University, Kent, Ohio, USA, 44242

[2] Liquid Crystal Institute, Kent State University, Kent, Ohio, USA, 44242

Video 1 and Video 2 show the imaging process from which Figure 3 (a-c) and (a'-c') have been obtained.

Video 1: "Video1.mov" shows images as the focus of the inverted microscope has been changes continuously from Object, LC in grid, and inverted image.

Video 2: "Video2. Mov" shows images as the focus of the inverted microscope has been changes continuously from the inverted image to the LC in grid (without polarizers, with left and right circular polarizers and in between green filter (between circular polarizers.

---

[1] Current affiliation: Physics and Materials Science Research Unit, University of Luxembourg, L-1511 Luxembourg, Grand Duchy of Luxembourg.
